# Safety assessment of genetically modified rice expressing Cry1Ab protein in Sprague–Dawley rats

**DOI:** 10.1038/s41598-021-80958-6

**Published:** 2021-01-13

**Authors:** Bahador Hajimohammadi, Gilda Eslami, Hengameh Zandi, Mohammad Hassan Ehrampoush, Azar Naimi, Maryam Derakhshan, Pegah Hedayat, Roozbeh Fallahi, Hossein Fallahzadeh, Mohammad Ebrahim Rezvani, Mahmoud Vakili, Seyed Mohammad Moshtaghioun, Seyyed Shamsadin Athari, Seyedeh Leili Asadi-Yousefabad, Saeedeh Sadat Hosseini, Mehrnoush Shirdeli, Salman Ahmadian, Shirin Mortazavi, Elahe Loni, Vahid Ajamein, Amin Ahmadi, Vahideh Askari

**Affiliations:** 1 Research Center for Food Hygiene and Safety, School of Public Health, Shahid Sadoughi University of Medical Sciences, Shohadaye Gomnam Blvd., Yazd, 8916188638 Islamic Republic of Iran; 2 Department of Pathology, Medical University of Isfahan, Isfahan, Iran; 3 Animal Viral Diseases Research Department, Razi Vaccine and Serum Research Institute, Agricultural Research Education and Extension Organization (AREEO), 3197619751 Karaj, Iran; 4 Department of Biostatistics and Epidemiology, Daneshjoo Boulevard, Health School, Shahid Sadoughi University of Medical Sciences, Shohadaye Gomnam Blv., Yazd, 8916188638 Islamic Republic of Iran; 5 Department of Physiology, School of Medicine, Shahid Sadoughi University of Medical Sciences, Shohadaye Gomnam Blvd., Yazd, 8916188638 Islamic Republic of Iran; 6 Department of Community and Preventive Medicine, Health Monitoring Research Center, Faculty of Medicine, Shahid Sadoughi University of Medical Sciences, Shohadaye Gomnam Blv., Yazd, 8916188638 Islamic Republic of Iran; 7 Department of Biology, Faculty of Sciences, Yazd University, Yazd, Iran; 8 Department of Immunology, School of Medicine, Zanjan University of Medical Sciences, Zanjan, Iran; 9 Department of Genetics, School of Medicine, Shahid Sadoughi University of Medical Sciences, Shohadaye Gomnam Blvd., Yazd, 8916188638 Islamic Republic of Iran; 10 Department of Food Science and Technology, Food Security Research Center, School of Nutrition and Food Science, Isfahan University of Medical Sciences, Isfahan, Iran; 11 Department of Pathobiology, Faculty of Veterinary Medicine, Ardakan University, Ardakan, Iran

**Keywords:** Biochemistry, Biotechnology, Plant sciences

## Abstract

Rice is considered one of the most important staple food crops. Genetically modified (GM) Bt rice, harbored *cry1Ab* gene expressing the insect-resistance protein has been developed to resistance to the insects. In this study, we assessed the safety of the GM Bt rice on Sprague–Dawley rats for 90 days. Totally, 120 rats in both sexes were used for three different diets, including 50% GM Bt rice, feeding with 50% rice, and standard feeding. Each 40 SD rats including 20 males and 20 females were considered as each diet. The clinical variables such as body weight and food consumption were measured and a range of clinical tests was examined, including hematology, serum chemistry parameters, urinalysis profile, thyroid, and sex hormone levels. Pathological assessments were also done. The results showed that the mean weekly feed utilization (%) had no significant difference among the studied groups. Also, blood biochemistry, hematological parameters, urine analysis, and hormonal levels had no significant differences among the groups. However, alanine aminotransferase was less in males versus female feeding with GM Bt rice. No histopathological changes were observed among the groups. In conclusion, this study demonstrated that GM Bt rice had no obvious adverse effects on rats' health.

## Introduction

Rice (*Oryza* *sativa* L.) is considered as one of the most important staple food crops cultivated in more than 120 countries^1^. It represents approximately 21% of all calories consumed by over 3 billion people all over the world^2^. This cereal crop is most severely damaged by insects. Various insects may invade all portions of the rice plant from root to panicle^3^ and annual losses of 10 million tons of rice are owed to insect-pest. Because of this problem, genetic engineering technology provides a new way to decrease the damage by insects. During the past 20 years, the successful production of genetically modified (GM) rice has been properly developed with insect resistance phenotype. These GM crops have the potential to significantly decrease the number of losses and can be used to enhance farm income^4^.

*Bacillus thuringiensis* (Bt) is known as a soil bacterium producing the insect-specific δ-endotoxins^5^. Since the mid-1980s, insect-resistance genes (*Bt* *cry*) have been transferred into plants^6^. So far, different types of *cry* genes have been cloned which express the insecticidal crystal proteins in GM foods, namely Cry1Ab^7^, Cry1Ac^8^, Cry1B^9^, Cry1C^10^, Cry1Aa^9^, Cry1Ca1^11^, Cry2A^12^, and Cry9C^13^. Among them, Cry1Ab toxin encoded by *cry1Ab* has high species-specific toxicity against stem borers (*Chilo suppressalis*)*.* The Bt *Tarom Molaii* rice line, an Iranian GM rice, was produced by insertion of the *Bt* *cry1Ab* gene that was cultivated in farms of the northern part of Iran at lab-scale to prevent the damages by *C. suppressalis*^14^. *Tarom Molaii* rice is a cultivar of isozyme group V which includes high-quality, low-tillering aromatic grains of rice of the Sadri and Basmati types. Transformation of *Tarom Molaii* through microprojectile bombardment by plasmid pCIB442, carrying *cryIAb* gene, enhanced resistance to stem borer^14^. *C.* *suppressalis* has a highly alkaline receptor on the gut epithelial cells that have a binding site for Cry1Ab^15^. The insecticidal activity of Bt protein is highly specific and occurs through receptors with the binding of the toxin resulting in pore formation and death of the insect^16^. It has been well documented that the Cry1Ab protein is nontoxic for mammals, probably due to the lack of specific receptors on mammalian gut epithelial cells^17^.

Like all GM crops, potential unintended effects must be evaluated to ensure the safety of GM foods. Although some researches have shown that Bt toxins (Cry proteins) are safe for consumption^18^, each event should be assessed for any potential risks. Therefore, the present study aimed at the safety assessment of the GM Bt *Tarom Molaii* rice during a 90-day feeding period in Sprague–Dawley (SD) rats.

## Methods

### Ethical statement

All experiments and animal housing procedures were ethically performed following standard protocols approved by the Ethics Committee of Shahid Sadoughi University of Medical Sciences, Yazd, Iran (Approval ID: IR. SSU. SPH.REC. 1395.39). The euthanasia procedure used in this study was based on AVMA Guidelines^19^ for the Euthanasia of Animals (AVMA, 2013) using 10:1 (mg/mg) solution of a ketamine-xylazine mix. We declare that all authors have read the checklist for ARRIVE guidelines (The ARRIVE guidelines 2.0: author checklist) and complied with its instructions.

### Animal diet preparation

According to our previous primary study^20^, the GM Bt rice (Ghareyazi et al. 1997; Event name: *Tarom Molaii*; no ID available) and non-GM near isoline rice were obtained from Agricultural Biotechnology Research Institute of Iran, Karaj, Iran. The feeds were prepared by the Razi Vaccine and Serum Research Institute, Karaj, Iran in three groups, including Group A: the standard feeds for rats with substitution of 50% carbohydrate with *Tarom Molaii* rice, Group B: the standard feeds for rats with substitution of 50% carbohydrate with GM Bt rice, and Group C: the standard feed.

### Animal and housing condition

The SD rats, including 60 male and 60 female with 3 to 4 weeks old were purchased from the Animal Research Center of the Razi Vaccine and Serum Research Institute, Karaj, Iran. The rats had the mean weight of 200 ± 20 g, which were randomly distributed in stainless steel wire cages as five rats per cage. The accessibility of feeds and water was ad libitum for all cages. The temperature of animal rooms was maintained at 23 ± 1 °C with the relative humidity of 55 ± 5%, 10 times/ h air change, and 12 h light/dark cycle. The feeding (with raw rice) duration was 90 days for all groups.

### Clinical observation

During the 90-day experimental period, clinical observations of the rats were conducted twice daily for mortality, abnormal signs, and unusual behaviors.

### Body weight, and mean weekly feed utilization

Bodyweight and food consumption were measured each week. Body weight, as well as food consumption were weekly measured for calculation of Mean weekly feed utilization (%) = (weekly one cage body weight gain/weekly one cage feed consumption) × 100.

### Sampling

After 90 days, urine and blood sampling was done. On the last day of the study, urine was gathered from each rat with the metabolic cage. Then, blood samples were taken from the aorta and collected in EDTA anticoagulation for hematological analysis and clot tubes for blood biochemistry assessment and hormone evaluation. At terminal sacrifice, each organ of the brain, spleen, liver, heart, uterus, ovary, testis, kidney, colon, thyroid, stomach, and esophagus was sampled for gross and histopathological examination.

### Blood biochemistry parameters

The serum was used for blood biochemical parameters analysis, including blood urea nitrogen (BUN), glucose (GLUC), cholesterol (Chol), triglyceride (TG), creatinine (Cr), high-density lipoproteins (HDL), alanine aminotransferase (ALT), aspartate aminotransferase (AST), total protein (TP), albumin (ALB), lactate dehydrogenase (LDH), calcium (Ca), bilirubin (Bili), and uric acid (UA). All analyses on blood serum were performed using an automatic biochemical analyzer, bs 380 (Mindray, China).

### Hematology

Hematology parameters were assessed using a hematology analyzer (Sysmex XP300, Japan), including platelets (PLT), white blood cells (WBC), red blood cells (RBC), hemoglobin (HGB), hematocrit (HCT), mean corpuscular volume (MCV), mean corpuscular hemoglobin (MCH), mean corpuscular hemoglobin concentration (MCHC), mean platelet volume (MPV), red cell distribution width (RDW), and platelet distribution width (PDW).

### Hormone levels

The serum samples were used for hormone analysis, including follicle-stimulating hormone (FSH), luteinizing hormone (LH), triiodothyronine (T3), thyroxine (T4), and thyroid-stimulating hormone (TSH) using ELISA.

### Urinalysis

The urine appearance of each rat was assessed. Dipstick (Bio Karpira, URS-10, Iran) was used for the analysis of pH, ketones (KET), urine specific gravity (USG), total protein (TP), urobilinogen (URO), blood, glucose, bilirubin (Bili), nitrite, and leukocytes. Microscopic examination was done using a light microscope (Olympus, CH2, Tokyo, Japan.

### Gross necropsy and histopathology

Complete gross necropsy was performed and all interested organs were prepared for pathological characterization. The organs were weighed, then sampling from the organs was done for histopathological assessment, including brain, heart, thyroid, intestine, stomach, liver, kidneys, spleen, esophagus, ovaries, testis, and uterus. The samples were stained with hematoxylin and eosin (H&E).

### Statistical analysis

All data were analyzed statistically using SPSS 16.0. A one-way analysis of variance (ANOVA) was applied to evaluate any significant differences among the groups. The post-hoc Tukey HSD test was used for any significant distances between the two groups. Differences were considered significant at *p* < 0.05.

## Results

### Physiology and clinical observation

During the 90-day feeding trial, all animals survived and no death occurred. Skin and fur of all rats were normal without any alopecia, cyanosis, and necrosis. Movements of rats were normal without any hyperactivity, hypersensitivity, paralysis, and prostrate. No secretion was seen from the eyes and no nasal discharge was reported. All the reflexes for each group were normal.

### Body weight and mean weekly feed utilization

**Figure 1:**
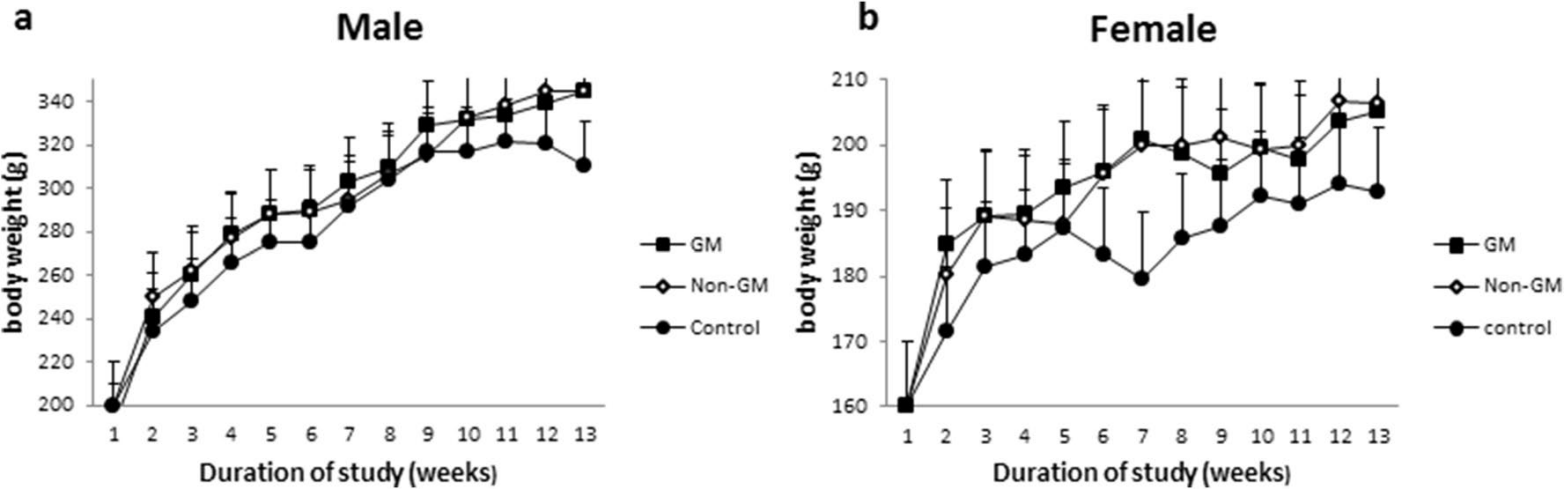
(**a**) Mean weekly body weight of male rats fed on different diets (**b**) Mean weekly body wight of female rats fed on different diets.

During the study, there were no statistically significant differences in the mean of weekly body weight gain for both FA (Female rats with *Tarom molaii* rice feeding) versus FB (Female rats with GM Bt rice feeding) (*P* = 0.803) and MA (Male rats with *Tarom molaii* rice feeding) versus MB (Male rats with GM Bt rice feeding) (*P* = 0.899). Growth curves are included for males and females in Fig. 1. Similar growth patterns for male rats were observed, although a reduction in body weight at weeks 6 and 7 for females was possibly due to stress, related to the unwanted and unknown factors. The mean weekly food utilization rate (%) had no significant differences among the studied groups (Table 1).

**Table 1:** Mean Food utilization rate (%) after feeding with different diets for 90 days in the studied groups.

Gender	Group	Mean food utilization rate (%)
Males	MA	7.4041 ± 4.101
	MB	8.3541 ± 5.111
	MC	8.1479 ± 8.261
Females	FA	3.90208 ± 4.119
	FB	2.5229 ± 2.163
	FC	1.86875 ± 2.314

Data are reported as mean ± SD. MA: male rats with *Tarom molaii* rice feeding, MB: male rats with GM Bt rice feeding, MC: male rats with standard feeding, FA: female rats with *Tarom molaii* rice feeding, FB: female rats with GM Bt rice feeding, FC: female rats with standard feeding.

### Blood biochemistry

The values of blood biochemistry parameters for all studied groups are shown in Table 2. No apparent differences were observed for all parameters except for ALT. The ALT had significant differences in FA versus FB (*p value* = 0.01) and FB versus FC (Female rats with standard feeding) (*p value = *0.001). The difference of ALT among all three groups of FA, FB, and FC was significant, too (*p value* = 0.0001).

**Table 2:** Serum biochemical parameters in the studied groups on the 90th day.

	Males			Females		
	MA	MB	MC	FA	FB	FC
GLUC (mg/dl)	233.39 ± 59.1	260.68 ± 40.64	254.39 ± 39.85	202 ± 33.99	198.42 ± 20.15	165.2 ± 36.88
BUN (mg/dl)	52.05 ± 7.56	46.53 ± 4.07	57.17 ± 9.38	52.85 ± 6.66	52.32 ± 7.13	62.4 ± 7.95
CREA (mg/dl)	0.85 ± 0.05	0.85 ± 0.05	0.82 ± 0.04	0.82 ± 0.06	0.84 ± 0.06	0.78 ± 0.05
TG (mg/dl)	49.95 ± 15.46	69.37 ± 40.3	47.33 ± 18.6	75.85 ± 23.81	73.95 ± 33.47	63.18 ± 29.6
Chol (mg/dl)	60.9 ± 9.2	56.95 ± 6.19	57.72 ± 9.46	66.35 ± 10.24	73.05 ± 10.2	69 ± 9.28
HDL (mg/dl)	35.85 ± 5.28	34.58 ± 6.41	35.61 ± 6.8	42.85 ± 7.67	46.82 ± 6.81	44.47 ± 7.79
ALT (IU/L)	76.1 ± 20.15	60.95 ± 11.67*	85.56 ± 21.96	73.95 ± 14.65	58.17 ± 14.96*	83.07 ± 18.23
AST (IU/L)	104.45 ± 18.8	104.37 ± 25.66	113.94 ± 13.82	118 ± 26.55	100.72 ± 17.52	134.47 ± 50.23
Ca (mg/dl)	9.57 ± 1.8	9.92 ± 0.96	9.71 ± 1.03	10.14 ± 0.44	10.14 ± 0.62	9.85 ± 0.56
Bili (mg/dl)	0.3 ± 0.0	0.3 ± 0.0	0.3 ± 0.0	0.3 ± 0.0	0.3 ± 0.0	0.3 ± 0.0
TP (mg/dl)	6.87 ± 0.7	6.5 ± 1.41	6.51 ± 1.7	7 ± 0.83	7.16 ± 0.62	7.01 ± 0.98
ALB (mg/dl)	2.91 ± 0.21	2.91 ± 0.21	2.85 ± 0.24	3.06 ± 0.27	3.24 ± 0.37	3.24 ± 0.37
LDH (mg/dl)	244.05 ± 354	271.33 ± 394.7	182.29 ± 40.96	269.88 ± 169.79	196.33 ± 72.09	190.54 ± 105.98
UA (mg/dl)	2.27 ± 1.89	1.9 ± 0.65	2.04 ± 0.68	2.03 ± 0.56	2.81 ± 0.88	2.44 ± 1.06

Each value is presented as mean ± SD. MA: male rats with *Tarom molaii* rice feeding, MB: male rats with GM Bt rice feeding, MC: male rats with standard feeding, FA: female rats with *Tarom molaii* rice feeding, FB: female rats with GM Bt rice feeding, FC: female rats with standard feeding. Gluc: glucose , BUN: blood urea nitrogen, CREA: creatinine, TG: triglyceride, Chol: cholesterol, HDL: high density lipoproteins, ALT: alanine aminotransferase, AST: aspartate aminotransferase, Ca: calcium, Bili: bilirubin, TP: total protein, ALB: albumin, LDH: lactate dehydrogenase, and UA: uric acid. No significant differences was observed in all groups but the ALT with the *p value* < 0.05.

### Hematology

The results of hematological parameters in all studied groups showed no statistically significant differences (*p* > 0.05; Table 3).

**Table 3:** Hematological parameters in the studied groups on the 90th day.

	Males			Females		
	MA	MB	MC	FA	FB	FC
WBC (10^3^/µl)	7.85 ± 3.06	7.13 ± 2.3	7.34 ± 4	6.48 ± 2	5.53 ± 1.2	5.35 ± 1.3
RBC (10^6^/µl)	8 ± 1.13	8.41 ± 0.46	8.15 ± 0.39	7.55 ± 0.3	6.92 ± 1.2	7.59 ± 0.2
HBG (g/dl)	12.9 ± 1.97	13.72 ± 0.54	13.52 ± 0.7	12.91 ± 0.45	12.31 ± 0.94	12.93 ± 0.54
HCT (%)	38.07 ± 5.41	40.64 ± 1.81	40.33 ± 1.7	38.14 ± 1.33	36.62 ± 2.47	38.23 ± 1.63
PLT (10^3^/µl)	717.5 ± 127.2	750.21 ± 64.63	729.39 ± 118.1	722.1 ± 75.92	685.67 ± 105.8	701.45 ± 105.32
MCV (fL)	47.66 ± 1.76	48.41 ± 1.45	49.57 ± 1.58	50.57 ± 0.9	51.03 ± 1.65	50.91 ± 2.48
MCH (pg)	16.66 ± 0.4	16.26 ± 0.39	16.54 ± 0.55	17.05 ± 0.4	16.97 ± 0.38	17.11 ± 0.61
MCHC (g/dl)	33.77 ± 0.74	33.72 ± 0.55	33.44 ± 0.64	33.79 ± 0.41	33.77 ± 0.47	33.61 ± 0.69
RDW (%)	14.17 ± 0.7	14.49 ± 0.55	14.38 ± 0.86	12.97 ± 0.8	13.39 ± 0.56	13.51 ± 0.92
PDW (fl)	14.31 ± 0.15	14.29 ± 0.55	14.24 ± 0.18	14.21 ± 0.17	14.17 ± 0.21	14.11 ± 0.21
MPV (fl)	8.38 ± 0.31	8.32 ± 0.27	8.12 ± 0.35	7.93 ± 0.17	7.88 ± 0.3	7.66 ± 0.29
PCT (%)	0.42 ± 0.27	0.55 ± 0.2	0.46 ± 0.22	0.57 ± 0.07	0.52 ± 0.15	0.51 ± 0.06

Each value is presented as mean ± SD. MA: male rats with *Tarom molaii* rice feeding, MB: male rats with GM Bt rice feeding, MC: male rats with standard feeding, FA: female rats with *Tarom molaii* rice feeding, FB: female rats with GM Bt rice feeding, FC: female rats with standard feeding. WBC: white blood cell, RBC, red blood cell; HGB, hemoglobin; HCT, hematocrit; PLT, platelet; MCV, mean cell volume; MCH, mean cell hemoglobin; MCHC, mean cell hemoglobin concentration; RDW, red blood cell distribution width; PDW, platelet distribution width; MPV, mean platelet volume; PCT, plateletcrit.

### Hormone levels

The data regarding the sexual and thyroidal hormones, including FSH (FA vs FB: *P* = 0.887) (MA vs MB: *P*  = 0.652), LH (FA vs FB: *P*  = 0.899) (MA vs MB: *P* = 0.610), T3 (FA vs FB: *P* = 0.286) (MA vs MB: *P* = 0.713), T4 (FA vs FB: *P* = 0.433) (MA vs MB: *P* = 0.899), and TSH (FA vs FB: *P* = 0.899) (MA vs MB: *P* = 0.899), using ELISA showed no significant differences in their levels in three various groups. The hormone levels are shown in Table 4.

**Table 4:** Hormonal analysis in the studied groups on the 90th day.

	Males			Females		
	MA	MB	MC	FA	FB	FC
FSH (IU/ml)	0.13 ± 0.24	0.05 ± 0.1	0.19 ± 0.41	0.36 ± 0.72	0.36 ± 0.34	0.5 ± 1.0
LH (IU/L)	0.3 ± 0.52	0.11 ± 0.14	0.4 ± 0.54	0.2 ± 0.31	0.32 ± 0.5	0.41 ± 0.98
T3 (ng/dl)	1.37 ± 0.43	1.45 ± 0.23	1.18 ± 0.38	1.38 ± 0.35	1.14 ± 0.4	1.27 ± 0.66
T4 (µg/dl)	4.38 ± 0.6	4.4 ± 0.61	4.4 ± 0.85	4.79 ± 0.79	5.13 ± 0.7	4.94 ± 0.98
TSH (IU/L)	0.07 ± 0.0	0.06 ± 0.1	0.17 ± 0.24	0.05 ± 0.0	0.05 ± 0.1	0.05 ± 0.0

Each value is presented as the mean ± SD. MA: male rats with *Tarom molaii* rice feeding, MB: male rats with GM Bt rice feeding, MC: male rats with standard feeding, FA: female rats with *Tarom molaii* rice feeding, FB: female rats with GM Bt rice feeding, FC: female rats with standard feeding. FSH: follicle stimulating hormone, LH: luteinize hormone, T3: triiodothyronine, T4: thyroxin, TSH: thyroid stimulating hormone.

### Urinalysis

As shown in Table 5, the urinalysis profile data showed no significant differences among the studied groups. Microscopic observation revealed that WBC and RBC were not shown in the urine of either group. Urine color in male and female rats was yellow in all samples and therefore no difference was seen between different groups. Also, urinary factors such as glucose, ketone, bilirubin, etc.were negative in all samples. Examination of urinary nitrite in male rats showed that it is only one of the rats with standard feeding had positive nitrite but it is not statistically significant.

**Table 5:** Urinalysis- Data profile from the studied groups on the 90th day.

Urinanalysis profile	Males			Females		
	MA	MB	MC	FA	FB	FC
pH	6.68 ± 0.98	6.65 ± 1	6.42 ± 0.75	7.08 ± 1.03	7.6 ± 1.2	6.63 ± 0.72
Appearance	clear	clear	clear	clear	clear	clear
Color	Yellow	Yellow	Yellow	Yellow	Yellow	Yellow
USG	1.014 ± 5.07	1.015 ± 9.86	1.014 ± 5.89	1.010 ± 5.91	1.009 ± 5.15	1.013 ± 9.23
URO (mg/dL)	ND	ND	0.5 ± 087	ND	0.11 ± 0.45	0.47 ± 1.09
KET	ND	ND	ND	ND	ND	ND
UTP	Trace	Trace	Trace	Trace	Trace	Trace
Leukocyte	ND	ND	ND	ND	ND	ND
Bilirubin	ND	ND	ND	ND	ND	ND
Nitrit	ND	ND	ND	ND	ND	ND
Glucose	ND	ND	ND	ND	ND	ND
Blood	ND	ND	ND	ND	ND	ND

ND = Not Detected. MA: male rats with *Tarom molaii* rice feeding, MB: male rats with GM Bt rice feeding, MC: male rats with standard feeding, FA: female rats with *Tarom molaii* rice feeding, FB: female rats with GM Bt rice feeding, FC: female rats with standard feeding. USG: urine specific gravity, URO: urobilinogen, KET: Ketones, UTP: urine total protein.

### Organ weights

Details regarding organ weights in all groups of MA (Male rats with *Tarom molaii* rice feeding), MB (Male rats with GM Bt rice feeding), MC (Male rats with standard feeding), FA, FB, and FC are summarized in Table 6. No significant differences in organ weights were observed among the studied groups.

**Table 6:** Organ weights of rats in the studied groups on the 90th day.

Organs (g)	Males			Females		
	MA	MB	MC	FA	FB	FC
Right kidney	1.52 ± 0.07	1.69 ± 0.12	1.55 ± 0.08	1.69 ± 0.12	1.03 ± 0.02	1.00 ± 0.04
Left kidney	1.59 ± 0.07	1.78 ± 0.14	1.48 ± 0.08	1.78 ± 0.14	1.04 ± 0.08	0.95 ± 0.02
Liver	11.98 ± 5.08	13.1 ± 5.57	12.25 ± 4.48	13.1 ± 5.57	7.59 ± 1.31	7.73 ± 0.6
Heart	1.16 ± 0.03	1.2 ± 0.04	1.13 ± 0.03	1.2 ± 0.04	0.79 ± 0.01	0.86 ± 0.23
Lung	1.58 ± 0.05	1.52 ± 0.18	1.62 ± 0.23	1.52 ± 0.18	1.14 ± 0.05	1.04 ± 0.04
Brain	1.91 ± 0.02	1.98 ± 0.02	1.96 ± 0.07	1.98 ± 0.02	1.82 ± 0.01	1.73 ± 0.01
Spleen	0.76 ± 0.04	0.76 ± 0.03	0.79 ± 0.02	0.76 ± 0.03	0.52 ± 0.01	0.63 ± 0.06
Right testis	1.65 ± 0.03	1.63 ± 0.01	1.6 ± 0.01	1.63 ± 0.01	–	–
Left testis	1.69 ± 0.03	1.73 ± 0.01	1.67 ± 0.01	1.73 ± 0.01	–	–

MA: male rats with *Tarom molaii* rice feeding, MB: male rats with GM Bt rice feeding, MC: male rats with standard feeding, FA: female rats with *Tarom molaii* rice feeding, FB: female rats with GM Bt rice feeding, FC: female rats with standard feeding.

### Gross necropsy and histopathology

**Figure 2:**
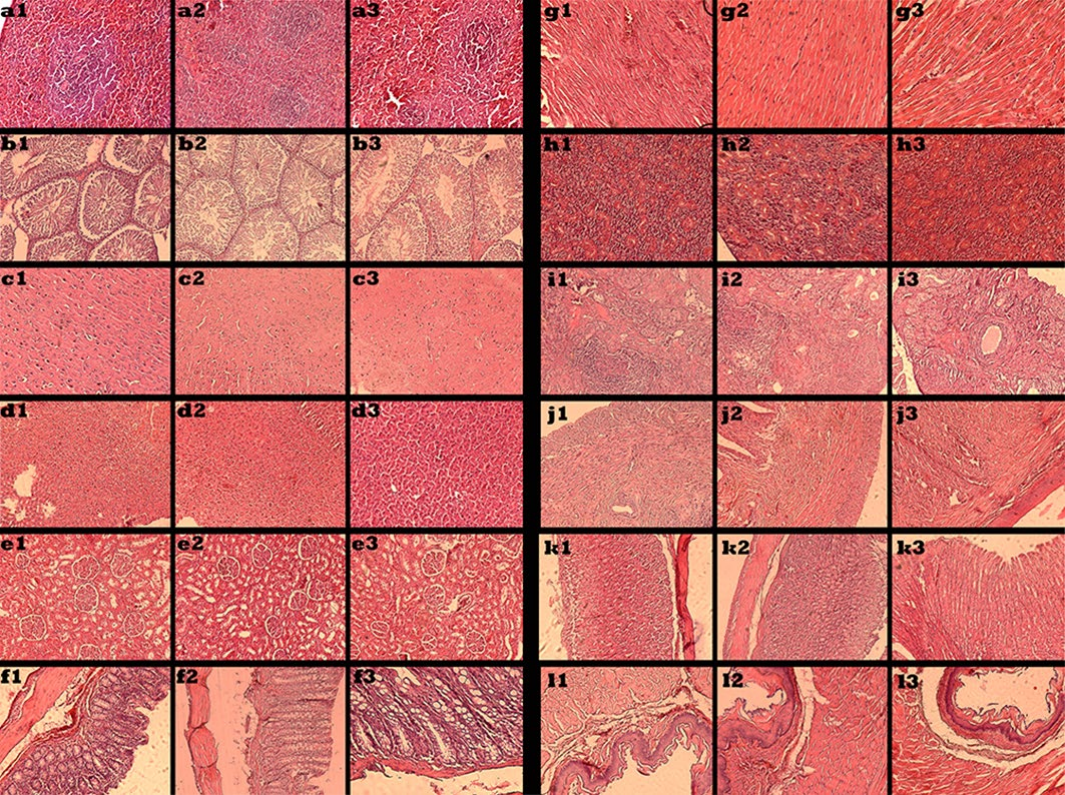
Tissue histopathology at 90 day, including spleen (**a1**: non-genetically modified diet, **a2**: genetically modified diet, **a3**: basic diet), testis (**b1**: non-genetically modified diet, **b2**: genetically modified diet, **b3**: basic diet), brain (**c1**: non-genetically modified diet, **c2**: genetically modified diet, **c3**: basic diet), liver (**d1**: non-genetically modified diet, **d2**: genetically modified diet, **d3**: basic diet), kidney (**e1**: non-genetically modified diet, **e2**: genetically modified diet, **e3**: basic diet), intestine (**f1**: non-genetically modified diet, **f2**: genetically modified diet, **f3**: basic diet), heart (**g1**: non-genetically modified diet, **g2**: genetically modified diet, **g3**: basic diet), thyroid (**h1**: non-genetically modified diet, **h2**: genetically modified diet, **h3**: basic diet), ovary (**i1**: non-genetically modified diet, **i2**: genetically modified diet, **i3**: basic diet), uterus (**j1**: non-genetically modified diet, **j2**: genetically modified diet, **j3**: basic diet), stomach (**k1**: non-genetically modified diet, **k2**: genetically modified diet, **k3**: basic diet), and esophagus (**l1**: non-genetically modified diet, **l2**: genetically modified diet, **l3**: basic diet). Tissues of rats stained with H&E.

During the complete gross necropsy analysis, no macroscopic pathological changes were found in all studied groups. Besides that, no atypical histopathological observations were presented for the brain, heart, thyroid, intestine, stomach, liver, kidneys, spleen, esophagus, ovaries, testis, and uterus in all groups (Fig. 2).

The liver and kidney are key metabolic organs which in our study, lobule structure of liver and arrangement were normal in all groups, and hepatocytes with no necrosis, normal nuclear and cytoplasm were observed. Also, portal spaces were with normal structure. In addition, the kidney has an important role in response to toxicants and we observed glomeruli and tubules with normal histology. Although mild congestion was found in two rats (MA and MB) but statistically was not significant.

This feeding study in SD rats demonstrated that in ovarian samples, lesions were not observed in follicle and corpus luteum, and stromal luteinization was found in group A and B. Single-layer endometrium with stroma surrounded by a muscular layer with normal histology was found in all groups of female rats. Also in male rats, the structure of seminiferous tubules was normal and testicular tissue with complete spermatogenesis was found.

The spleen as an important center of immunological responses has normal histology of white pulp and red pulp and no differences were observed between group B vs A and C. Pathological findings in other tissues, including the heart, brain, thyroid, and intestine were as follows:

Heart: In all three feeding groups, the normal structure of cardiac muscles with clear cross striations can be seen and nuclear was in the center.

Brain: Cerebrum and cerebellum with normal histology were found.

Thyroid: Follicles with normal histology and some glandular structure with cells having eosinophilic cytoplasm were observed.

Gasterointestinal tissue: In intestine, edema and lymphoplasma cell and neutrophilic infiltration in lamina propria was seen in group B, but not other two groups.

In addition to the above, the stomach and esophagus were seen with normal histology of muscle structure.

Generally, during the analysis of all tissues, observed changes were not statistically significant.

## Discussion

Food production in response to population growth is one of the most important issues all over the world. Recently, biotech crops that are resistant to major insect pests have been demonstrated with their potential to significantly decrease the loss amounts^21^. The GM Bt *Tarom Molaii* rice has been generated for this target. Animals are appropriate goals for medical researches, including developing vaccines, drugs, and toxic studies^22^. The present study was the first safety assessment and hematological, biochemical, physiological parameters analysis as well as histopathological assessing for GM Bt *Tarom Molaii* rice on SD rats for 90 days.

Our results showed that no sign of toxicity or adverse effects were found in the studied groups. As these parameters are regarding so many factors such as age, environment, and genetic factors, therefore in this study all mentioned factors were considered identical for all studied groups but the diet types. Blood and urine sampling was done just after the investigation period and therefore the condition was stressless for all animals. All of the rules by NC3Rs were observed for all groups resulting in minimal stress. Therefore, it seems that the data would be similar to the real items and reflected the direct effect of nutrients that is the only variable item among the three groups. In this regard, it was revealed that the GM Bt rice caused no obvious adverse effects on rats as evaluated by several biological parameters, including organ weight, serum chemistry, hematology, thyroidal and sexual hormones level, urinalysis, and histopathology. Few studies have been conducted on the safety assessment of Bt rice. Yuan et al. found no toxicity in SD rats fed by GM T2A-1 rice^23^. Also, similar findings have been reported by Zhou who observed no adverse health. They evaluated the effect of GM rice line (TRS) on SD rats through three generations and the results indicated that differences were not biologically meaningful^22^.

In our research, to blood biochemistry, significantly lower levels of ALT were observed in both sexes from the GM groups in comparison with non-GM and also control groups. Based on the study by Delwatta^24^ the ALT (IU/L) in SD rats is very wide with the median of 1–223.3 in females and 2.1–426.9 in males. It is well-known that the level of ALT is firmly related to some kind of effects on liver and AST/ALT ratio which is a guide to determine fibrosis in liver disease. However, no effects on liver weight and, no histopathological findings were observed. Therefore, the decreased ALT level in comparison with the one in the other groups may be biologically considered insignificant. On the other hand, the cholesterol level that could be the indication of liver damage was similar in the experiment and control group. Results from some studies suggested that more folate-rich foods have hepatoprotective effect in animal models and consuming it to diet linked to lower serum ALT levels^25^, but based on the compositional analysis GM Bt rice is similar to its near conventional counterpart^15^. Poulsen investigated the safety of GM rice expressing PHA-E lectin on Wistar rats and found that changes in ALT activity could be a sign of liver damage. However, they found no histopathological change. These researchers claimed that an increased level of ALT is probably due to the systemic metabolic disturbance caused by the PHA-E lectin^26^. Also, in a comprehensive study carried out by Song^27^on SD rats, the AST/ALT ratio was similar in the GM group (mfb-MH86 line) and non-GM group and there were no gross pathological findings.

Differences in food intake can affect the growth and physiological parameters of the body and the change in the food intake can also reflect in a change of body weight. In the current study, no differences were observed in food intake and also body weight in different groups. This phenomenon suggests that the organ development of the rat can not be influenced by taking in the GM Bt *Tarom Molaii* rice for a 90-day period which is consistent with the finding of Wang^28^. These researchers indicated that Cry1Ab protein has no effect on the organ weight of Swiss rats between the group with the diet of GM rice (Shanyou 63) and control groups which are per our findings. Additionally, all the hematology and serum chemistry data from the present research, urinalysis profile, serum sex hormones, and thyroid hormones level were similar in rats on the GM Bt *Tarom Molaii* rice diet, the near-isogenic rice diet and the control diet which are similar to the results of the study carried out by Zhou, et al*.*^2^. These authors revealed that serum sex hormone levels of both male and female SD rats fed by GM high amylose rice were not statistically different from those in the control groups.

The histopathological examination revealed no corresponding adverse effects and no changes to the adverse direction. Schroder clearly showed that in a 90-day feeding study on Wistar rats, both differential count and pathology of immune organs like thymus and spleen indicated that reduced amount of white blood cells (WBC) observed in the test group, which fed with the GM KMD1 rice expressing Cry1Ab protein, is biologically insignificant^15^. This suggests that changes in pathological findings are important keys for the recognition of differences between the groups, but we revealed no atypical or group-related histopathological observations. In the study carried out by Tang, et al.^29^, SD rats fed GM rice T2A-1 and no gross pathological findings were found during necropsy that is in agreement with our study. Mao, et al. have recently used cynomolgus macaques as an animal model, because of their similarity to humans in physiology. They designed a 52-week feeding trial for Bt rice line Huahui 1 (HH1) and found no adverse or toxic effects of Bt rice on biochemistry and pathological parameters^30^. Therefore, this study is in the agreement with our study.

In this study, three different diet groups were analyzed which can provide a more clear comparison between the probable toxicological effects of each feeding group. Also, unlike some studies, we had an appropriate sample size to have a safety assessment study for GM Bt rice that is one of the main advantages of our study. To increase the specificity and sensitivity of the study, an additional test group which should be fed with pure recombinant Cry1Ab protein and different concentrations of GM rice which formulated into rodent diets is also suggested to detect the specific compound-related effects. It is important to emphasize that the event in used this study was GM Bt *Tarom Molaii* rice and therefore, the present results can not be generalized to the other GM rice events. In conclusion, we evaluated the toxicological effects of GM Bt *Tarom Molaii* rice during a 90-day feeding period in SD rats. It can be concluded that GM Bt rice showed no unintended obvious adverse effect on the health of rats. To obtain more comprehensive data, safety of this GM Bt rice should be evaluated in the other laboratorial animals in the next feeding trials in future.
